# Low prevalence targets are primarily missed due to mind wandering

**DOI:** 10.3758/s13414-026-03296-7

**Published:** 2026-06-29

**Authors:** Haden Dewis, Michael C. Hout, Joseph W. Houpt, Lois Howsley, Hayward J. Godwin

**Affiliations:** 1 School of Psychology, University of Southampton, Shackleton Building, Highfield Campus, Southampton, SO17 1BJ UK; 2 Department of Psychology, New Mexico State University, Las Cruces, NM USA; 3 Department of Kinesiology, New Mexico State University, Las Cruces, NM USA; 4 Department of Psychology, The University of Texas at San Antonio, San Antonio, TX USA

**Keywords:** Mind wandering, Visual search, Low prevalence effect

## Abstract

The impact of target prevalence (the proportion of trials in which a target appears) has been widely studied within visual search. The low prevalence effect is the finding that rare targets are often missed. Mind wandering, where one’s attention drifts away from a primary goal, impairs performance across various tasks, but its influence in low-prevalence search remains unclear. We recruited 60 participants from the general population and examined their mind-wandering rates as they searched for either low- (10%) or high- (50%) prevalence targets. When participants were engaged in mind wandering, response accuracy was reduced and target-absent response times increased. Low-prevalence targets were missed more than high-prevalence targets, but to our surprise, we discovered that most of the low-prevalence effect could be attributed to mind wandering in the majority our analyses. This marks a substantial shift in our understanding of the prevalence effect.

## Introduction

Target prevalence refers to the proportion of trials wherein a target is presented, and a consistent finding is that when targets appear rarely (e.g., 1% of trials), they are more likely to be missed than when they appear often (e.g., 50% of trials; Wolfe et al., 2005). Many important real-world search tasks such as baggage screening at airports and radiology require searches for low-prevalence targets. As such, over the last several decades, a large portion of the search literature has aimed to better understand the effects of low target prevalence on behavior and performance. The causes, consequences, and solutions to the prevalence effect have been examined in many different experiments (Fleck & Mitroff, 2007; Godwin et al., 2015, 2016; Hout et al., 2015; Wolfe et al., 2007; Wolfe & van Wert, 2010). Here, we offer a new account of the prevalence effect, based on the hypothesis that when target prevalence is low, searchers mentally disengage from the task more often, and when that happens, targets become more likely to be missed.

There has been extensive theorizing regarding the prevalence effect. Early work suggested that reductions in target prevalence give rise to a criterion shift in decision making, such that searchers are biased towards responding “absent” on each trial (Wolfe et al., 2007). More recent research has utilized eye tracking to further understand why low-prevalence targets are missed (Godwin et al., 2015; Hout et al., 2015). These studies have led to the understanding that low-prevalence targets are missed for multiple reasons: low-prevalence targets are often missed because they are not directly examined (i.e., fixated upon) and in the rare instances when these targets are fixated, they are more likely than high-prevalence targets to be erroneously rejected as distractors and thereby missed by searchers.

To date, past research has not yet examined whether mind wandering contributes to the effects of low-target prevalence. Mind wandering involves one’s mind drifting away from a primary task, towards unrelated, inner thoughts, feelings, or fantasies (Smallwood & Schooler, 2006). This is extremely common; most people will experience it many times every day. For our present purposes, we refer to mind wandering as a situation wherein an individual’s attention is no longer being focused on the primary task/goal at hand.^1^ Research has shown that mind wandering is associated with poor performance across a wide range of different cognitive tasks (see Mooneyham & Schooler, 2013, for a review). As such, we expected that long “runs” of target-absent trials would become tedious for participants, and therefore become likely to induce episodes of mind wandering, as seen in prior research (Kane et al., 2017; Randall et al., 2014; Smallwood et al., 2004).

To our knowledge, no previous research has determined how much of the prevalence effect can be attributed to mind wandering. We addressed this directly with a study wherein participants were asked to search for T shapes amongst a set of L shapes with either a low level of prevalence (10%) or a high level of prevalence (50%). Following standard mind-wandering study protocols (e.g., McVay & Kane, 2012; Smallwood & Schooler, 2006), we periodically asked participants whether they were mind wandering during the previous trial. Post-trial accuracy feedback was not given to participants to prevent them from realizing any connection between mind wandering and erroneous responses.

We made several key predictions. First, we predicted that mind-wandering rates would be higher in low- versus high-prevalence search. This is because boredom is known to increase mind wandering and long stretches of target-absent trials likely become monotonous or boring for participants. Second, since mind wandering negatively impacts performance within cognitive tasks (e.g., Mooneyham & Schooler, 2013), we predicted that accuracy rates would decrease when participants were mind wandering. Third, we predicted that response times (RTs) would increase when participants were mind wandering due to disengagement from the task that would not otherwise normally occur. Finally, as evidence that the prevalence effect is at least partly due to mind wandering, we predicted that the effects of target prevalence upon our measures could be explained to some degree by mind wandering (i.e., that mind wandering would mediate the effects of low target prevalence).

## Method

### Power analyses

We conducted two experiments. The first served as a proof-of-concept and the second as a higher-powered replication of the first. Due to the underpowered nature of the first experiment, we are not reporting directly on its methods or findings; instead, these can be found via the Open Science Framework at: https://osf.io/y748c/. Power analyses were conducted using the *simr* package in R (Green & MacLeod, 2016) with the data collected from the first experiment. These power analyses revealed a sample size of ~50 participants with 350 mind-wandering probe trials per participant required to allow all key interactions to have a power level of at least 80%. Therefore, we set the prevalence of mind-wandering probes at 25% and the number of search trials at 1,420.

### Participants

Participants were recruited using the online research platform *Prolific.com*. We advertised the study only to participants who reported themselves to be in the UK and to be fluent speakers of English. We also limited the study to be advertised only to those who had 95% or above approval ratings (i.e., 95% or more of each participants’ prior datasets had been approved by researchers as being of high quality). All participants provided informed consent prior to starting the experiment, which was approved by the Ethics Committee at the University of Southampton, ERGO 73512.A1. A total of 71 participants (age: *M* = 40.73, *SD* = 12.39 years; sex: female = 57.14%, male = 42.86%) were recruited and paid £12.00 for taking part.

### Apparatus

Stimuli were presented via participants’ own computer screens on a browser of their choosing. In order to take part, participants’ screens needed to be a minimum of 800 × 600 pixels. If their screens were smaller than this, the experiment would not let them proceed. Participants indicated responses via the “Z” and “M” keys. For the visual search trials, the “Z” key indicated a target-absent response, and the “M” key indicated a target-present response. During mind-wandering probes, the “Z” key was used to indicate on-task thoughts, and the “M” key to indicate off-task thoughts.

### Stimuli

Stimuli consisted of “T” and “L” shapes. Participants were asked to search for a target “T” shape of a specific color amongst a set of distractor “L” shapes on each trial. The target color was selected at random from a set of 16 colors that have been used in previous search studies (Godwin et al., 2015, 2016; Menneer et al., 2007; Stroud et al., 2012). Each distractor was randomly assigned one of these 16 different colors. The selected target color did not change throughout the course of the experiment.

On each trial, a total of 16 objects were selected at random and placed on a virtual 5 × 4 grid before being “jittered” by a random distance of up to 20 pixels away from their start point on the *x* and *y* planes. Each object was also rotated randomly by increments of 90° (i.e., 0°, 90°, 180°, 270°). Each trial contained either 15 non-target objects and one target object (target-present trial) or 16 non-target objects (target-absent trial). The number of target-present/absent trials each participant received depended on which target prevalence group they were assigned to. Those in the low-prevalence condition completed 142 target-present trials and 1,278 target-absent trials, whilst those in the high-prevalence condition completed 710 target-present and 710 target-absent trials.

### Design and procedure

Participants were randomly assigned to either the low-prevalence condition (the target appeared on 10% of the trials) or the high-prevalence condition (the target appeared on 50% of the trials). Following standard procedures in mind-wandering studies (McVay & Kane, 2009, 2012; Smallwood et al., 2004; Smallwood & Schooler, 2006), we assessed mind wandering using a series of *probe trials*. During these probe trials, participants were asked to select whether they were “thinking about the search task” or “thinking about something else” on the previous search trial. As a means of encouraging participants to be honest in their responses, they were told the following before starting the study – “Please do be as honest as possible. If you were not thinking about the search task, that is not a problem. After all, we all engage in mind wandering every day!” Probe trials followed 25% of the visual search trials and occurred on both target-present and target-absent trials.

Compared to other mind-wandering work (e.g., McVay & Kane, 2009, 2012; Smallwood et al., 2004; Smallwood & Schooler, 2006), a probe rate of 25% is quite large. Our rationale for this specifically relates to low prevalence. To study the impact of mind wandering on low-prevalence target detection, it was necessary to obtain data from target-present trials where mind wandering had also occurred. In the low-prevalence condition, targets appeared on only 10% of trials. This drastically reduced the number of trials where both the target was present and the searcher was engaged in mind wandering. As such, we substantially increased the rate of mind-wandering probes to address this bottleneck.

**Fig. 1:**
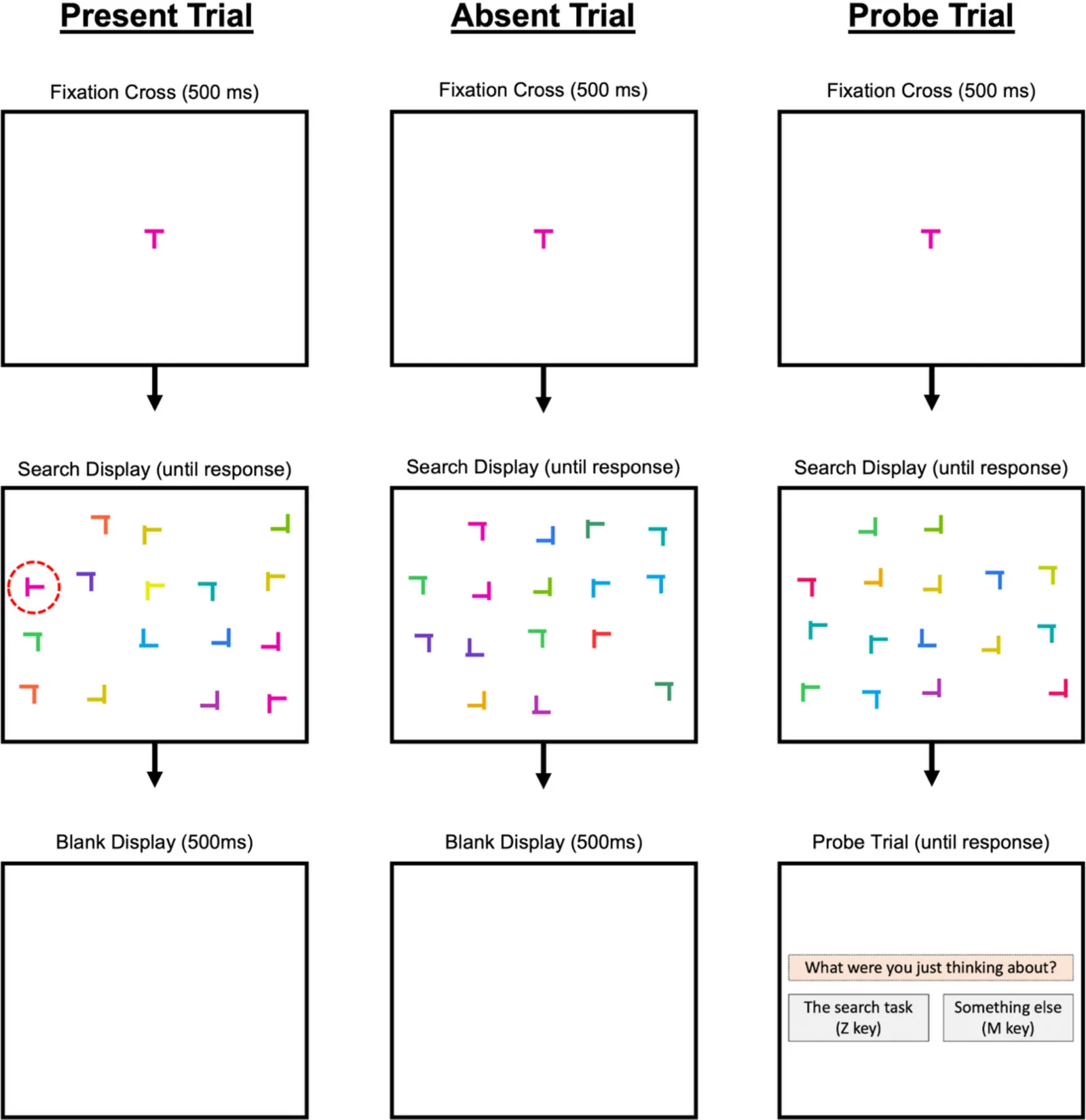
Sequence of Events in the Search Trials and Probe Trials. The left figure depicts a target-present search trial without a mind-wandering probe, center figure depicts a target-absent search trial without a mind-wandering probe, and right figure shows a search trial followed by a mind-wandering probe. Non-targets were varied in color, rotation, and location across trials, whereas the target (highlighted by a red dashed circle) was only varied in rotation and location. The red dashed circle was not included in the real experiment and is only included here to aid target visibility

Participants began by taking part in 20 practice trials. These were followed by 1,420 search trials. Probe trials were randomized such that one probe occurred at random within “windows” of four trials, with the constraint that no two consecutive trials could contain a probe. No feedback was given to participants following responses. This was key to ensure participant responses were not a result of demand characteristics, i.e., responding that they were mind wandering following an incorrect search trial. A summary of the trial procedure can be seen in Fig. 1. Each trial began with a fixation cross for 500 ms. The display then appeared until a response had been made. Following a response, a blank screen appeared for 500 ms.

## Results

### Data preparation

Before beginning our analyses, we applied preplanned cleaning procedures that were similar to those used in prior online search task research (Dewis et al., 2025; Godwin et al., 2024; Godwin & Hout, 2023). Our reason for not using identical cleaning procedures to those used in the previously cited studies was simply due to differences in the study design. For example, our study did not include eye tracking, and as such, it was not possible to completely replicate their approaches, for example, removing guessing trials – trials where the participant responded present yet never fixated the target. Instead, inclusion was based on a set of four criteria. First, participants who did not complete all trials within their respective experiment were removed. The remaining criteria were based on participants’ RTs. The aim here was to only include participants who effortfully engaged in the task. Participants who spent longer than 60,000 ms on any single trial were removed. Following this, any participants who had more than five trials shorter than 250 ms or five trials longer than 20,000 ms were removed from the dataset. The breakdown of the total number of participants removed is in Table 1. Following cleaning, the final datasets consisted of 85,137 search trials and 21,105 probe trials from 60 participants (33 in the high-prevalence condition, 27 in the low-prevalence condition).

**Table 1:** Data-cleaning steps and associated removals

Cleaning stage	No. of participants removed	No. of trials removed	Total participants remaining	Total trials remaining
Raw data	0 (0.00%)	0 (0.00%)	71 (100%)	100,820 (100%)
Did not finish experiment	0 (0.00%)	0 (0.00%)	71 (100%)	100,820 (100%)
Had trials longer than 60,000 ms	7 (9.86%)	9,940 (9.86%)	64 (90.14%)	90,880 (90.14%)
More than five fast trials	4 (5.63%)	5,680 (5.63%)	60 (84.51%)	85,137 (84.51%)
More than five slow trials	0 (0.00%)	0 (0.00%)	60 (84.51%)	85,137 (84.51%)

Fast trials = < 250 ms, Slow trials = > 20,000 ms. The table depicts the number and percentage of participants removed for each inclusion criteria level. Final step removes any individual fast or slow trials that might remain

### Analytic approach

We examined effects using hierarchical Bayesian logistic regression models for accuracy measures, and Bayesian shifted log-normal regression models for RT measures, through the software package Stan (Gabry et al., 2025). There is disagreement within the literature regarding the optimal approach to use when interpreting such models and model fits (i.e., Gronau & Wagenmakers, 2019; Vehtari et al., 2019). As such, for the sake of transparency and to avoid cherry-picking results that agree with our predictions, we interpreted our results in three distinct steps.

In step one we examined the posterior distribution of the relevant regression coefficients to determine whether confidence intervals (CIs) passed through zero. Then, across the next two steps, we assessed model fits with and without relevant parameters to find the “best fitting” model for our data, using two related metrics. The first of these metrics was Bayes factors (BFs). The BF is a likelihood ratio test between contrasting hypotheses, for example, an alternative and a null hypothesis. BFs greater than 1.00 suggest stronger evidence for the alternative hypothesis whilst BFs less than 1.00 indicate stronger evidence for the null hypothesis. The second of these metrics was the expected log pointwise predictive density difference (ELPD). The ELPD is a measure that quantifies the difference in predictive accuracy between two models, where values smaller than 0.00 favor the second model. We have categorized differences by magnitudes of standard error within ELPD scores. For example, if the ELPD difference is greater than 2 × the standard error of difference (SE diff), then this is likely a reliable difference between model types.

As is the case within multiverse analyses (for a discussion of these in the context of visual search, see Godwin et al., 2021), we have based our conclusions upon findings consistent across each of these three steps. If an effect was present within each of these steps, then we could be confident that it was a reliable finding. Likewise, if an effect was present in only one of these steps, then the reliability of that effect was less certain.

### Confirming the prevalence effect

We started by confirming that the prevalence manipulation worked as intended. For the sake of brevity, we have reported only the main findings from our hierarchical models of response accuracy and RTs. The remaining model comparisons can be found at https://osf.io/y748c/.

When modelling accuracy, none of the posterior CIs of relevant parameters included zero. The interaction between Target-Presence and Prevalence was positive (*β* = 0.55, CI = [0.46, 0.64]), indicating that accuracy was relatively higher on target-present trials when prevalence was high; however, accuracy was lower (*β* = −1.71, CI = [−1.81, −1.62]) for target-present trials compared to target-absent trials, and low prevalence was associated with lower accuracy overall (*β* = −0.20, CI = [−0.33, −0.08]). In summary, there was evidence in favor of low prevalence leading to lower accuracy in target-present trials.

For correct RTs, the posterior CIs did not include zero for any of the relevant parameters. Target-present trials were associated with faster RTs (*β* = −0.14, CI = [−0.18, −0.10]), and high target prevalence was associated with slower RTs overall (*β* = 0.11, CI = [0.02, 0.19]). Finally, the interaction between Target-Presence and Prevalence was negative (*β* = −0.17, CI = [−0.21, −0.14]), indicating that RTs were faster on target-present trials when prevalence was high compared to when prevalence was low. Put simply, there was evidence in favor of lower prevalence leading to faster responses on target-absent trials.

Overall, then, the full analyses revealed that the standard effects of target prevalence emerged (i.e., targets were more likely to be missed in the low-prevalence condition and RTs for target-absent trials were briefer in low prevalence).

### Examining the effects of mind wandering

**Fig. 2:**
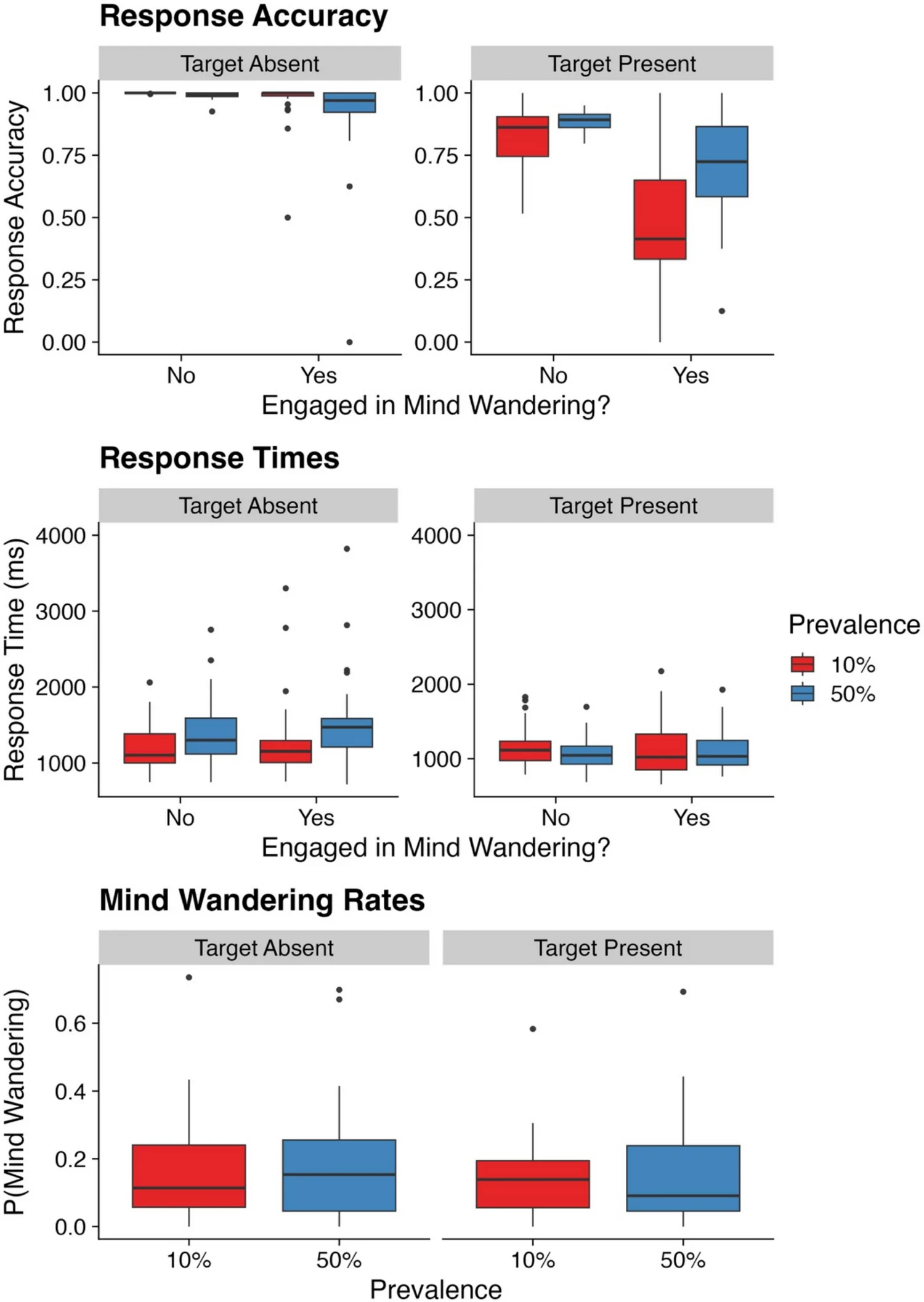
Response accuracy, response time, and mind wandering rates. Whiskers show the range of values within quartile 3+1.5×Interquartile range to quartile 1–1.5×interquartile range. Horizontal black lines indicate the median

**Fig. 3:**
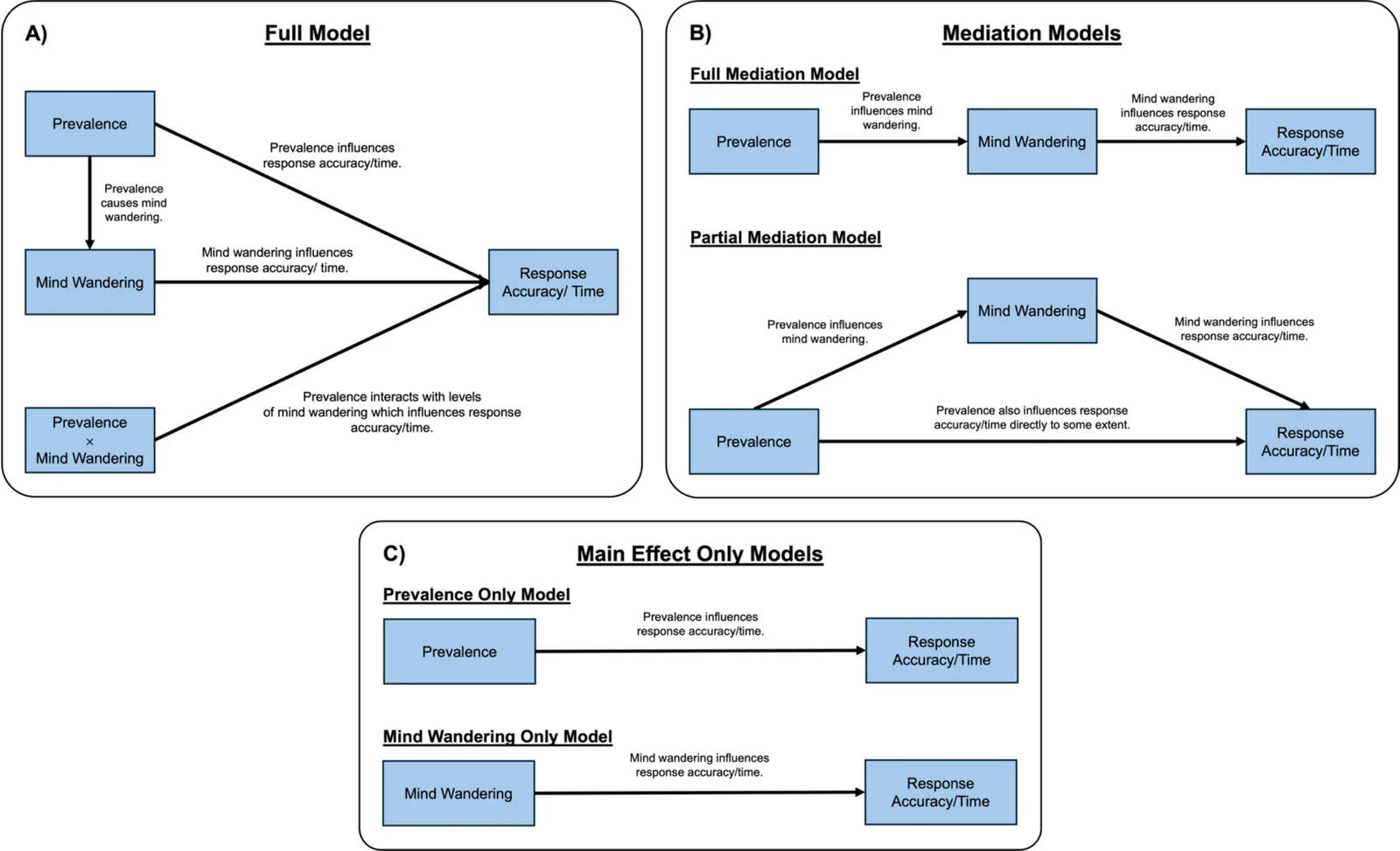
Model depictions. The figure depicts all models used for model comparisons (excluding null models) within the context of our experiments. Panel **A** depicts the full model including all effects, panel **B** depicts the mediation models, both full and partial, and panel **C** depicts models where main effects only were modeled

Next, to assess the relationship between mind wandering and response accuracy, we used hierarchical Bayesian logistic regression models as predicted by Prevalence, Mind Wandering, and the interaction between the two, along with an indirect influence of Prevalence on response accuracy via Mind Wandering (i.e., a partial mediation model). For modelling RTs, we used a shifted log-normal regression model with the same structure of independent variables. To verify effects, we both examined the posterior distribution of the relevant regression coefficients and assessed the model fits with and without relevant parameters using BFs and ELPDs. Descriptive statistics for all remaining analyses can be found within Fig. 2, BFs and ELPDs for all model comparisons can be found within Table 2, and visual depiction of models can be found within Fig. 3.

**Table 2:** Examining the effects of mind wandering – model comparisons

Model type	Model notation	Response accuracy				Response time			
		Target present		Target absent		Target present		Target absent	
		*BF*_*10*_	*ELPD*	*BF*_*10*_	*ELPD*	*BF*_*10*_	*ELPD*	*BF*_*10*_	*ELPD*
Full	${{p}_{i}\sim \mathrm{logistic}\left({\mu}_{s}+{b}_{p}\cdot {Prevalence}_{i}+{b}_{m}\cdot {MW}_{i}+{b}_{m\times p}\cdot {Prevalence}_{i}\cdot {MW}_{i}\right)}{{MW}_{i}=logistic\left({\mu}_{m,s}+{\gamma}_{p}\cdot Prevalence\right)}$	1/8.10	−0.30 (2.40)	1/4.80	−1.10 (0.60)	<1/89	−17.0 (3.00)	<1/10^14^	<−48.00
Partial Mediation	${p}_{i}\sim \mathrm{logistic}\left({\mu}_{s}+{b}_{p}\cdot {Prevalence}_{i}+{b}_{m}\cdot {MW}_{i}\right){{MW}_{i}=logistic\left({\mu}_{m,s}+{\gamma}_{p}\cdot Prevalence\right)}$	1/9.60	−0.70 (1.90)	1/4.40	0.00	1/9.40	−2.70 (1.60)	1/1.07	−0.02 (0.70)
Full mediation	${p}_{i}\sim \mathrm{logistic}\left({\mu}_{s}+{b}_{m}\cdot {MW}_{i}\right){{MW}_{i}=logistic\left({\mu}_{m,s}+{\gamma}_{p}\cdot Prevalence\right)}$	1/9.90	−1.80 (1.00)	1.00	−0.08 (2.00)	1/9.10	−1.70 (2.70)	1/1.04	0.00
No prevalence	${p}_{i}\sim \mathrm{logistic}\left({\mu}_{s}+{b}_{m}\cdot {MW}_{i}\right){{MW}_{i}=logistic\left({\mu}_{m,s}\right)}$	1.00	0.00	1/1.60	−1.50 (2.00)	1.00	0.00	1.00	−0.02 (0.80)
No mind wandering	${p}_{i}\sim \mathrm{logistic}\left({\mu}_{s}+{b}_{p}\cdot {Prevalence}_{i}\right)$	<1/10^3^	<−90	<1/10^14^	<−30	<1/89	−3.30 (3.10)	<1/10^14^	<−48
Null	${p}_{i}\sim \mathrm{l}\mathrm{o}\mathrm{g}\mathrm{i}\mathrm{s}\mathrm{t}\mathrm{i}\mathrm{c}\left({\mu}_{s}\right)$	<1/10^3^	<−90	<1/10^14^	<−30	<1/89	−1.60 (3.00)	<1/10^14^	<−48

The table depicts model notation for all models compared and their associated Bayes factors (BFs) and Expected Log Pointwise Predictive Density Difference (ELPD). Values within parentheses represent the standard error difference

The common priors used within the models were as follows:

$${\mu}_{m,s}\sim {\mathrm{Normal}}\left(\mathrm{0,1}\right)$$

$${\mu}_{g}\sim Normal\left(\mathrm{0,1}\right)$$

$$R\sim LKJ\left(2\right)$$

$$\upsigma \sim {\mathrm{Exponential}}\left(1\right)$$

$${b}_{m,g}\sim {\mathrm{Normal}}\left(\mathrm{0,1}\right)$$

$$\left[\begin{array}{c}{\mu}_{s}\\ {b}_{m,s}\end{array}\right]\sim {\mathrm{MVNormal}}\left(\left[\begin{array}{c}{\mu}_{g}\\ {b}_{m,g}\end{array}\right],S\right)$$

$${\gamma}_{p}\sim {\mathrm{Normal}}\left(\mathrm{0,1}\right)$$

$${b}_{p}\sim {\mathrm{Normal}}\left(\mathrm{0,1}\right)$$

$${b}_{m\times p}\sim {\mathrm{Normal}}\left(\mathrm{0,1}\right)$$

$${\mathrm{MW}}_{i}=\left\{\begin{array}{lc}{\mathrm{m}\mathrm{w}}_{i}, \ \ \ \ \ \ \ \ \ \ \ \ \ \ \ \ \ \ \ \ \ \ \ \ \ \ \ \ \ \ \ \ \ \ \ \ \ if\ probed\\ {\mathrm{logistic}}\left({\mu}_{m,s}+{\gamma}_{p}\cdot {\mathrm{Prevalence}}\right), \ \ otherwise\end{array}\right.$$

$${p}_{i}\sim \mathrm{logistic}\left({\mu}_{s}+{b}_{p}\cdot {\mathrm{Prevalence}}_{i}+{b}_{m}\cdot {\mathrm{MW}}_{i}+{b}_{m\times p}\cdot {\mathrm{Prevalence}}_{i}\cdot {\mathrm{MW}}_{i}\right)$$

$${\mathrm{Mind-wandering}}_{i} \sim {\mathrm{Bernoulli}}\left({\mathrm{MW}}_{i}\right)$$

$${\mathrm{Accuracy}}_{i} \sim {\mathrm{Bernoulli}}\left({p}_{i}\right)$$

### Accuracy and mind-wandering rates: Probe-target-present trials only

We began by examining the influence of target prevalence and mind wandering on response accuracy for target-present trials. Overall, we found an interaction between low target prevalence and mind wandering across response accuracy within target-present trials. Here, as shown within the top-right panel of Fig. 2, when mind wandering, response accuracy was substantially worse for those in the low-prevalence compared to those within the high-prevalence condition.

Within our hierarchical model, the posterior CIs did not include zero for any relevant parameter: the main effect of Mind Wandering was negative (*β* = −0.72, CI = [−0.83, −0.60]), indicating a decrease in accuracy overall when engaged in mind wandering; the main effect of Prevalence was positive (*β* = 0.58, CI = [0.37, 0.80]), indicating increased accuracy within the higher prevalence condition; the Mind Wandering × Prevalence interaction was positive (*β* = 0.16, CI = [0.05, 0.27]), thus indicating that when engaged in mind wandering, those within the high-prevalence condition displayed greater accuracy than those within the low-prevalence condition; Finally, the effect of Prevalence on Mind Wandering was negative (*β* = −0.44, CI = [−0.66, −0.22]), indicating that high-prevalence trials resulted in less mind wandering. Overall, this analysis suggested an interacting effect of both mind wandering and target prevalence on response accuracy, and that as predicted, target prevalence increased rates of mind wandering.

#### Bayes factors

Model comparison using BFs indicated that the model with an effect of Mind Wandering but no effect of Prevalence on either accuracy or mind wandering rates was the best-fitting model. The BFs for this model were better than they were for the full model including interactions (*BF*_*10*_ = 1/8.10), the model with all main effects (partial mediation; *BF*_*10*_ = 1/9.60), and the model with mind wandering fully mediating the prevalence effect (*BF*_*10*_ = 1/9.90). The remaining models had much lower relative scores (*BF*_*10*_ < 1/10^3^). Overall, this analysis therefore suggested that reductions in accuracy were predominately a result of mind wandering over target prevalence.

#### ELPDs

When using ELPD, we found the same results as we did for the BFs above. The ELPD favored the model with an effect of Mind Wandering but no effect of Prevalence on either response accuracy or mind-wandering rates. The ELPD for this model was better than it was for the full model including interactions (*ELPD* = −0.30, *SE_DIFF* = 2.40), the model with all main effects (partial mediation; *ELPD* = −0.70, *SE_DIFF* = 1.90), and the model with mind wandering fully mediating the prevalence effect (*ELPD* = −1.80, *SE_DIFF* = 1.00). Likewise, the remaining models had much lower relative scores (*ELPD* < −90.00). Overall, as before, our ELPDs again suggested that reductions in accuracy were predominately a result of mind wandering over target prevalence.

#### Summary

Overall, across all the different approaches we found that changes within response accuracy rates were primarily a result of mind wandering, although there may also be an additional, smaller, direct effect of prevalence. Our hierarchical model suggested that interaction between Prevalence and Mind Wandering contributed to decreases in response accuracy. However, our model comparisons suggested that these negative effects on response accuracy were instead sufficiently explained by mind wandering alone. In other words, the direct effect of target prevalence itself upon response accuracy in target-present trials was, to our surprise, very weak indeed.

### Accuracy and mind-wandering rates: Probe-target-absent trials

Next, we turned to target-absent trials and again analyzed effects across three distinct steps. Within the first step, the posterior CIs did not include zero for the direct effect of Prevalence (*β* = −0.71, CI = [−1.05, −0.36]), nor for the effect of Mind Wandering (*β* = −0.72, CI = −0.91, −0.53]), thus indicating a decrease in accuracy for target-absent trials both independently when target-prevalence was high and when participants were engaged in mind wandering. However, the CIs did include zero for both the interaction term (*β* = 0.02, CI = [−0.16, 0.19]) and the effect of Prevalence on mind-wandering rates (*β* = −0.11, *CI* = −0.30, 0.11]). Simply put, we observed an increase in false alarms within target-absent trials when target-prevalence was high and when participants were engaged in mind wandering. However, in contrast to our predictions, there was not a clear indication of an effect of target prevalence upon mind wandering rates within target-absent trials.

#### Bayes factors

When using BFs, the best-fitting model was that which included Prevalence as being fully mediated by Mind Wandering. However, there was not a large difference between this and the models that included a main effect of Mind Wandering only (*BF*_*10*_ = 1/1.60), main effects of both Prevalence and Mind Wandering only (partial mediation; *BF*_*10*_ = 1/4.40), or the full model including interactions (*BF*_*10*_ = 1/4.80). Overall, this suggested that any observed effects of target prevalence on response accuracy were primarily the result of target prevalence influencing mind wandering.

#### ELPD

When comparing models using ELPD, the favored model was that which included both the main effects of Prevalence and Mind Wandering on accuracy, and the main effect of Prevalence on mind-wandering rates, but no interaction (i.e., partial mediation). However, there was not a large difference between this model and the model containing all effects and interactions (*ELPD* = −1.10, *SE_DIFF* = 0.60), nor the full mediation model (*ELPD* = −0.08, *SE_DIFF* = 2.00), nor the model with an effect of Mind Wandering only (*ELPD* = −1.50, *SE_DIFF* = 2.00). Overall, this would suggest that similar to BFs, the observed influence of target prevalence on response accuracy was predominately due to target prevalence influencing mind wandering but an additional direct effect of prevalence and an interaction effect are plausible.

#### Summary

Overall, our model comparisons suggested that whilst low target prevalence appeared to influence response accuracy within target-absent trials, this effect was again somewhat weak and appeared to be primarily driven by changes in mind wandering. These findings align neatly with our target-present analyses.

### Response times and mind wandering rates: Probe-target-present trials only

Next, we focused on RTs. For correct target-present RTs, the posterior CIs of our model included zero for all relevant parameters except for the effect of Prevalence on mind-wandering rates (*β* = −0.45, CI = [−0.67, −0.23]). Here, our modelled effects indicated a reduction in mind wandering when Prevalence was high for correct target-present trials. The main effects of Mind Wandering (*β* = 0.02, CI = [−0.01, 0.05]), and Prevalence (*β* = 0.02, CI = [−0.06, 0.11]) were close to zero as was the interaction between Mind Wandering and Prevalence (*β* = 0.01, CI = [−0.02, 0.04]). In other words, this analysis showed no effects of Prevalence or Mind Wandering on RTs. It did, however, suggest that mind-wandering rates were influenced by target-prevalence.

#### Bayes factors

When comparing models, the BFs favored the Mind Wandering-only model with “substantial” evidence over the full mediation model (*BF*_*10*_ = 1/9.10) and partial mediation model (*BF*_*10*_ = 1/9.40), and stronger evidence against the remaining models (*BF*_*10*_ = 1/89.00 and lower). Overall, this analysis suggests that within our dataset, mind wandering was the predominant driver of variation within RTs for correct target-present trials.

#### ELPDs

Likewise, when examining ELPDs, the favored model was the one that only included an effect of Mind Wandering (and no indirect effects of Prevalence via Mind Wandering), with both the null model (*ELPD* = −1.60, *SE_DIFF* = 3.00) and the model where Prevalence was fully mediated by Mind Wandering (*ELPD* = −1.70, *SE_DIFF* = 2.70) as close runners-up. Overall, in line with the previous BFs, this analysis suggested that, again, RT variations for correct target-present trials were best explained as a result of mind wandering and not target prevalence.

#### Summary

When examining correct target-present trials only, target-prevalence likely had no effect on RTs but may influence rates of mind wandering. In line with this, and our previous accuracy analyses, our model comparisons emphasized that variations within correct target-present RTs were best explained as being a result of mostly Mind Wandering.

### Response times and mind-wandering rates: Probe-target-absent trials only

Lastly, we turn to correct RTs for target-absent trials. Here, the posterior CIs for the Mind Wandering coefficient (*β* = 0.02, CI = [0.01, 0.04]) and Prevalence coefficient (*β* = 0.11, CI = [0.00, 0.22]) did not include zero, indicating an increase in RTs when one was either engaged in mind wandering or exposed to higher target-prevalence. Both intervals for the effect of Prevalence on mind-wandering rates (*β* = −0.11, CI = [−0.32, 0.11]) and the interaction between Mind Wandering and Prevalence (*β* = 0.01, CI = [−0.01, 0.03]) included zero. Overall, this analysis suggested that both increases in target prevalence and increases in mind wandering resulted in longer target-absent RTs.

#### Bayes factors

The BFs for our model comparisons favored the model with an effect of Mind Wandering only, and no effect of Prevalence on mind wandering rates. However, this model was not clearly distinguished from the model that included both Prevalence and Mind Wandering effects (partial mediation; *BF*_*10*_ = 1/1.07), nor the full mediation model (*BF*_*10*_ = 1/1.04). Relative to all other models, the BFs were on the order of 10^14^ or higher. Overall, this analysis suggested that, as was the case within our response accuracy analyses, effects of target-prevalence on target-absent RTs were weak and were instead best explained as being predominantly mediated by changes in mind wandering.

#### ELPDs

When comparing models using ELPD, the favored model was the one in which the effect of Prevalence was fully mediated by Mind Wandering. However, the model containing only an effect of Mind Wandering and no effect of Prevalence on mind-wandering rates (*ELPD* = −0.02, *SE_DIFF* = 0.80) and the model with effects of both Prevalence and Mind Wandering (partial mediation; *ELPD* −0.02, *SE_DIFF* = 0.70) were approximately the same in terms of ELPD. The remaining models had an ELPD of −48.00 or higher with *SE_DIFF* of around 9.00. As was the case for the previously mentioned BFs, this analysis suggested that any effects of target prevalence on target-absent RTs were instead better explained as being a result of changes in mind wandering when prevalence was low.

#### Summary

In summary, RTs for correct target-absent trials appeared to increase both when target prevalence was high compared to when it was low, and when one was engaged in mind wandering versus being focused on the task. Our model comparisons suggested that these effects were once again largely a result of mind wandering, either via mediation of Prevalence or via Mind Wandering directly.

### Key findings

Overall, we have observed a set of consistent results. First, when examining response accuracy, due to a combination of both target-prevalence and mind wandering, we observed increases in miss rates for target-present trials and increases in false-alarm rates for target-absent trials. Second, when examining RTs, we found no effects of target prevalence or mind wandering on the time taken to respond within target-present trials. However, within target-absent trials, RTs increased both when Prevalence was high and when one was engaged in mind wandering.

A key factor that became clear across all analyses was that we did not observe particularly strong effects of target prevalence. In fact, according to most of our model comparisons, any effects of target prevalence were often better explained by mind wandering. In other words, once having measured mind wandering, keeping track of target prevalence often did not add any additional information for predicting response accuracy or RTs within our models.

## Discussion

The effects of target prevalence in visual search have been extensively studied (for a review, see Donnelly et al., 2019), but to date, no links have been made between low target prevalence and mind wandering. Here, we tested a simple question: to what extent does mind wandering contribute to the prevalence effect? We addressed this question by incorporating mind-wandering probes into a simple visual search task wherein target prevalence was manipulated. Throughout the study, participants were periodically asked whether they were mind wandering on the previous trial.

We observed a surprising set of results. First, we predicted that mind-wandering rates would be greater in low versus high prevalence search. Indeed, this was an effect that we observed across several of our analyses. Second, we predicted reductions within response accuracy rates when participants were engaged in mind wandering. In line with this, we observed this effect across both target-present and target-absent trials. Third, we predicted that RTs would increase when participants were mind wandering due to disengagement from the task; however, we only observed this within target-absent trials. Finally, we predicted that the effects of target prevalence upon both measures could be explained, at least in part, by mind-wandering rates (i.e., partial mediation). Indeed, in line with our expectations, this was something we observed, and as will now be discussed, we believe best explains our findings.

Many of our models suggested that changes in response accuracy and RTs were best explained solely by mind wandering without considering the target prevalence. We had anticipated that mind wandering could explain *some* of the prevalence effect – that is why we conducted this experiment after all – but we did not expect that mind wandering could explain *all* of the prevalence effect. This is an extreme stance to take and would imply that the prevalence effect does not exist in the form that is has been studied for the past two decades.

Rather than conclude that the prevalence effect can be explained by mind wandering alone, we instead draw a more conservative conclusion: that the effects of prevalence can be very much explained by mind wandering, but not *only* by mind wandering. We make this more conservative conclusion for several reasons. The first is that within our analyses, whilst the results indicated that prevalence had little or no explanatory value once mind wandering was accounted for, for some subsets of the data, that evidence was weaker. The models with no prevalence or an indirect effect of prevalence via mind wandering only were the best in the model comparisons, but usually by only a small margin. As such, the evidence is not unequivocal that the prevalence effect can be explained by mind wandering alone. The second is that it would be difficult to reconcile the idea that the prevalence effect is caused by mind wandering and nothing else with the substantive existing work on this topic. With that in mind, we conclude that that the most likely outcome was that target prevalence influenced mind wandering, mind wandering influenced our outcome measures, and that prevalence also had some direct effect on our outcome measures (as illustrated in panel B of Fig. 3).

Future work will need to tease apart this issue in more detail, but it is clear that we need to engage in closer scrutiny of the prevalence effect and its causes. For now, we note several important points relating to our results and possible future studies. First, we expect that we are *underestimating* the effects that mind wandering has upon the prevalence effect. This is based on the idea that the act of asking participants if they were mind wandering may have reduced their mind-wandering rates by refocusing their attention (Weinstein, 2018). As such, had we not probed regarding mind wandering during trials, the true rate of mind wandering may have been much higher. Indeed, replication of the current experiment with and without mind-wandering probes to assess the magnitude of influence probing has on the prevalence effect would be beneficial. Second, we used simple stimuli in our experiment. Even when mind wandering, participants often detected the targets. Had we used more complex real-world stimuli, then we would anticipate that the effects of mind wandering may differ. Detecting a T shape whilst one’s mind is elsewhere may be straightforward, but detecting a complex hidden weapon in a piece of simulated luggage may be a far more difficult task (e.g., see the stimuli used in Godwin et al., 2024). In other words, the extent to which different search scenarios (e.g., Godwin et al., 2010; Hout et al., 2017) may either increase, or protect from, the effects of mind wandering remains unclear. These points suggest that future work would benefit from using alternative methods, such as eye tracking or psychophysiological techniques to determine when participants are mind wandering, rather than asking them directly, as well as utilizing a host of different stimuli and search paradigms to test mind-wandering rates in more ecologically valid tasks as well.

What does mind wandering do to search behavior when prevalence is low? Our analyses of target-absent RTs suggest that, when mind wandering, search is slowed as participants disengage from the task. This is interesting because eye-movement studies have often found that searchers take longer to both first fixate targets in low prevalence and take longer to identify rare targets when they are fixated (Godwin et al., 2015; Hout et al., 2015). Perhaps it is the case that mind wandering draws information processing resources away from the search task, causing search behavior to be both slower and less accurate. Such disengagement could also explain the rise in the likelihood that searchers will fixate targets and still fail to detect them (Godwin et al., 2015; Hout et al., 2015), in what are now termed “look but fail to see” errors (Wolfe et al., 2022).

In summary, we examined to what extent mind wandering contributed to the prevalence effect. We are the first to show evidence of mind wandering playing a causal factor in the low prevalence effect within visual search. To our surprise, we discovered that much of the low prevalence effect could be attributed to mind wandering in most of our analyses. As such, it is important that current theories of visual search be revised, and future models incorporate these findings to accurately explain visual search behavior.

## Data Availability

All data from our experiments are publicly available on the Open Science Framework at https://osf.io/y748c/. Materials for this experiment have not been made available.

## Footnotes

1 It is important to differentiate between mind wandering during visual search and vigilance. In vigilance studies, observers spend long periods of time monitoring displays for subtle-to-detect events (Hancock, 2013). Though at first glance these two paradigms appear similar, there are some important differences, as reviewed by Wolfe et al. (2007): (1) in vigilance studies, targets appear and disappear without participants necessarily responding, whilst in search studies, stimuli remain visible until a response; (2) in vigilance studies, stimuli are typically simple; and, most importantly, (3) the same targets are frequently missed by different participants when prevalence is low, supporting the idea that a criterion shift is a core contributor to the prevalence effect, as opposed to random lapses of vigilance. As such, effects observed in the vigilance literature are not necessarily directly analogous to the effects observed in low-prevalence visual search tasks.

## Code availability

All code used for processing, analyzing, and visualizing data for this article is available on the Open Science Framework at https://osf.io/y748c/.

## Declarations

### Artificial intelligence

No artificial intelligence assisted technologies were used in this research or the creation of this article.
